# PREVALENCE AND ASSOCIATED RISK FACTORS OF HEPATITIS B VIRUS INFECTION IN LAFIA METROPOLIS, NASARAWA STATE, NIGERIA

**DOI:** 10.21010/Ajidv19i1.6

**Published:** 2024-10-25

**Authors:** ANGBALAGA Gladys Abel, SHOHAIMI Shamarina, MOHD NADZIR Mohd Noor Hisham, AB RAHMAN Abdul Hafiz

**Affiliations:** 1 Department of Biology, Faculty of Science, Universiti Putra Malaysia, 43400 UPM Serdang, Selangor, Malaysia; 2 Department of Microbiology, Federal University of Lafia, Nasarawa State, P.M.B 146, Nigeria; 3 Center for Research in Development, Social and Environment, Faculty of Social Sciences and Humanities, Universiti Kebangsaan Malaysia

**Keywords:** Hepatitis B, Nasarawa, Nigeria, Prevalence, Risk factors

## Abstract

**Background:**

Hepatitis B (HB), caused by the hepatitis B virus (HBV), remains a critical public health challenge in Nigeria. Globally, the HBV infects approximately 296 million people, significantly contributing to morbidity and mortality, with liver cirrhosis and liver cancer ranking as the 11th and 24th leading causes of death, respectively. This study aimed to determine the prevalence of HBV infection and the associated risk factors within the Lafia Metropolis, Nasarawa State, Nigeria.

**Materials and Methods:**

A cross-sectional study was conducted from September to November 2023, utilizing a validated questionnaire to assess 461 randomly selected participants from four communities (Azuba, Bukan Sidi, Danka Sarki, and Doka), representing diverse sociodemographic profiles and varying degrees of exposure to risk factors associated with HBV infection. Data were analyzed using chi-square (χ^2^) tests with IBM SPSS statistical software version 28.0 at a significance level of p < 0.05.

**Results:**

The findings showed that the overall prevalence of HBV infection was 7.8%. A significant association was observed between the prevalence of HBV infection and monthly income (p < 0.01). However, no associations were found with age, gender, educational level, marital status, occupation, or religion. Regarding risk factors, a significant association was found between HBV infection and awareness of one’s HBV status (p < 0.03).

**Conclusion:**

These findings highlight the need for targeted public health interventions and policies aimed at reducing HBV transmission among high-risk sociodemographic groups in Lafia Metropolis. This approach could potentially reduce the burden of HBV and improve health outcomes in affected communities.

## Introduction

Hepatitis B (HB) is a serious liver infection primarily transmitted through direct contact with infected blood or bodily fluids (Rajamoorthy *et al.*, 2019; Suan *et al.*, 2019). The hepatitis B virus (HBV) replicates in liver cells (hepatocytes), causing damage and potentially leading to death (Adeyemi *et al.*, 2013; Ullah *et al.*, 2022). HBV infection was previously ranked as the tenth leading cause of death worldwide, but currently, liver cirrhosis and liver cancer are the 11th and 24th leading causes, respectively (Devarbhavi *et al.*, 2023).

Nearly one-third of the global population have serological evidence of infection, either past or current, and approximately 296 million people are currently infected, including children under the age of five. This results in nearly 820,000 deaths annually associated with HBV diseases (Bigna *et al.*, 2019; Lingani *et al.*, 2018). In the Western Pacific Region and Africa, the prevalence of HBV infection is recorded at 6.2% and 6.1%, respectively (Khazaei *et al.*, 2018). In Nigeria, the prevalence rate varies between 12.2% and 14% across different sub-regions of the country (Olayinka *et al.*, 2016; Oti *et al.*, 2021), with reported rates of 7.1% and 13.3% in Nasarawa State. High-endemic regions tend to experience increased morbidity and mortality (Dwiartama *et al.*, 2022; Ndubuisi *et al.*, 2022). Therefore, this study was conducted to determine the prevalence and associated risk factors of HBV infection in Lafia Metropolis, Nasarawa State, Nigeria, and to explore which sociodemographic group is primarily affected.

## Materials and Methods

### Study design and location

Nasarawa State is one of the 36 states in Nigeria. Lafia, the state capital, is one of the 13 local government areas of the state. Lafia is located at 8.480^°^ North latitude and 8.520^°^ East longitude, with an elevation of 290 meters above sea level and a land area of 27,117 km^2^. Nasarawa State and its surroundings are in Nigeria’s middle belt (north-central region), characterized by an average temperature of 31 ^°^C and an average humidity of 64% (Angbalaga *et al.*, 2023).

**Figure 1:**
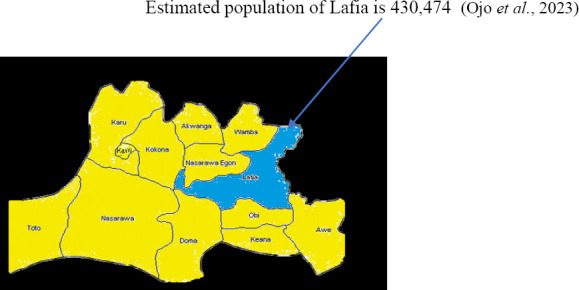
Map of Nasarawa state showing the study area.

### Data source

A questionnaire was designed, validated, and distributed to obtain data on exposure and outcomes from consenting participants in the study area. Following the questionnaire administration, blood samples were collected from the participants for testing. The completed questionnaires were used for analysis.

### Questionnaire design

Information concerning HBV infection was obtained by administering questionnaires to the respondents and testing the participants. Data collection was conducted using a self-structured, closed-ended questionnaire comprising two sections. Section A consisted of sociodemographic factors, and Section B consisted of risk factors for the respondent.

### Questionnaire development

The study questionnaire was developed from various articles (Abere *et al.*, 2022; Anka *et al.*, 2016; Rajamoorthy *et al.*, 2020; Roien *et al.*, 2021) to meet the study’s objectives. A panel of medical experts moderated the questionnaire, leading to modifications in the wording and organization of some questions. A pilot test was conducted based on feedback from respondents, which informed the final version of the questionnaire.

### Questionnaire validation

The validation process ensured that the questionnaire accurately measured the intended variables, which is a critical criterion in research. The questionnaire underwent a thorough validation approach, including face validity, construct validity, content validity, and reliability testing (Almanasreh, Moles, and Chen, 2019; Binti Daud *et al.*, 2021; Park *et al.*, 2018). The final questionnaire was confirmed to be readable and easy to understand according to the Flesch reading ease test value and the Flesch-Kincaid grade level test score. Based on established guidelines, these measures indicated good content validity, internal consistency, and test-retest reliability (Namdeo and Rout, 2016).

### Sample size determination and sample collection

The sample size was calculated using a Raosoft sample size calculator at a 95% confidence level with a 5% margin of error, resulting in a minimum sample size of 384, which was increased by 20% to a total of 461 participants (Pourhoseingholi, Vahedi, and Rahimzadeh, 2013). Four communities (Azuba, Bukan Sidi, Danka Sarki, and Doka) were enrolled in the study to ensure diverse representation of sociodemographic characteristics, which enable a comprehensive assessment of the associated risk factors and transmission dynamics across different segments of the population. These communities have different sociodemographic profiles, healthcare access, population density, and potential exposure to risk factors associated with hepatitis B virus infection. A cross-sectional study was conducted with the involvement of community heads from September to November 2023, and only consenting participants were recruited. A random sampling technique was used to distribute the questionnaire (Berger and Zhang, 2005), and honesty was emphasized to the participants. All questionnaires were anonymous, with codes assigned to them. A trained medical laboratory scientist from the Beacon Youth Initiative (BYI) NGO, Lafia, collected a 2 mL blood sample from each participant, and the resulting serum was tested for HBsAg using rapid test strips (ACON Laboratories, Inc., USA).

### Host-associated risk factors

Host-associated risk factors refer to variables that increase the likelihood of transmitting the infection within a given population. These factors were collected using the validated questionnaire.

### Data analysis and presentation

Statistical analysis was performed using the Statistical Product for Service Solution (SPSS) and Microsoft Excel. Appropriate statistical methods were applied to all quantitative data based on the type and distribution (McHugh, 2013; Van Belle & Fisher, 2004). The chi-square (χ^2^) test was used for analysis at a 95% confidence level and a 5% level of significance (p< 0.05) for all analyses (Connelly, 2019; Nihan, 2020). IBM SPSS statistical software version 28.0 was used to analyze the data and associations between the variables were determined.

### Ethical Considerations

The study was granted approval by the Nasarawa State Ministry of Health through the Ethical Committee, dated May 15, 2023, with NHREC Protocol No. 18/06/2017RA19462. Unless the respondent specifically requested otherwise, each respondent provided informed consent, as participation was voluntary. Records from the screening and information obtained from the questionnaires were treated with strict confidentiality and utilized solely for this research purpose.

## Results

**Table 1:** Prevalence of hepatitis B infection among the population of Lafia

Community	No. examined	No. Infected (%)	p-value
Azuba	116 (25.2)	11 (9.5)	
Bukan Sidi	115 (24.9)	11 (9.6)	
Danka Sarki	115 (24.9)	6 (5.2)	
Doka	115 (24.9)	8 (7.0)	
Total	461 (100.0)	36 (7.8)	**(χ^2^= 2, df = 3, p = 0.57)**

**Table 2:** Sociodemographic factors among the population of Lafia

Parameters	Group	Infected count n (%)	Uninfected count n (%)	No. examined n (%)	χ^2^	df	p-value
**Age of Respondents**	<10 10-19	2 (3.3) 6 (8.0)	58 (96.7) 69 (92.0)	60 (13.1) 75 (16.3)	4.66	5	0.46
	20-29	10 (7.9)	116 (92.1)	126 (27.3)			
	30-39	9 (10.3)	78 (89.7)	87 (18.9)			
	40-49	6 (12.2)	43 (87.8)	49 (10.6)			
	>50	3 (4.7)	61 (95.3)	64 (13.9)			
**Gender of Respondent**	Male	21 (8.4)	230 (91.6)	251 (54.4)	0.24	1	0.63
	Female	15 (7.1)	195 (92.9)	210 (45.6)			
**Educational level**	Non-formal	11 (6.6)	155 (93.4)	166 (36.0)	8.48	4	0.07
	Primary	4 (3.3)	116 (96.7)	120 (26.0)			
	Secondary	14 (12.5)	98 (87.5)	112 (24.3)			
	Degree	7 (11.7)	53 (88.3)	60 (13.0)			
	Masters	0 (0.0)	3 (100.0)	3 (0.7)			
**Marital status**	Single	17 (8.6)	180 (91.4)	197 (42.7)	0.62	3	0.89
	Married	18 (7.3)	229 (92.7)	247 (53.6)			
	Divorced/Separated	0 (0.0)	4 (100.0)	4 (0.9)			
	Widow/Widower	1 (7.7)	12 (92.3)	13 (2.8)			
**Occupational status**	Student	6 (4.8)	118 (95.2)	124 (26.9)	4.71	5	0.45
	Unemployed	6 (6.8)	82 (93.2)	88 (19.0)			
	Housewife	3 (6.1)	46 (93.9)	49 (10.6)			
	Business	14 (9.7)	130 (90.3)	144 (31.2)			
	Private	4 (10.8)	33 (89.2)	37 (8.0)			
	Public servant	3 (15.8)	16 (84.2)	19 (4.1)			
**Religion**	Islam	20 (6.9)	269 (93.1)	289 (62.7)	0.85	1	0.36
	Christian	16 (9.3)	156 (90.7)	172 (37.3)			
**Monthly income (N)**	< 20,000	23 (7.0)	306 (93.0)	329 (71.4)	13.31	4	0.01
	20,000-50,000	9 (9.7)	84 (90.3)	93 (20.2)			
	51,000-100,000	3 (9.7)	28 (90.3)	31 (6.7)			
	101,000-150,000	0 (0.0)	7 (100.0)	7 (1.5)			
	≥ 151,000 & above	1 (100.0)	0 (0.0)	1 (0.2)			

**Table 3:** Prevalence of HBV in association with Risk Factors The distribution of various risk factors among the respondents was determined by their HBV infection status, as recorded below:

Parameter	Infected count n (%) Yes No	Uninfected count n (%) Yes No	χ^2^	df	p-value
Do you have history of liver disease in your family*	11 (2.39) 25 (5.42)	75 (16.27) 350 (75.92)	3.64	1	0.06
Have you ever lived with HBV infected person*	7 (1.52) 29 (6.29)	78 (16.92) 347(75.27)	0.03	1	0.87
Do you know your HBV status*	13 (2.82) 23 (4.99)	88 (19.09) 337 (73.10)	4.60	1	0.03
Do you have a history of blood transfusion*	3 (0.65) 33 (7.16)	55 (11.93) 370 (80.26)	0.64	1	0.42
Have you had any contact with infected blood/bodily fluid*	5 (1.08) 31 (6.72)	48 (10.41) 377(81.78)	0.22	1	0.64
Have you travelled recently to HBV endemic region*	3 (0.65) 33 (7.16)	26 (5.64) 399(86.55)	0.28	1	0.60

## Discussion

### Prevalence among the population of Lafia

The prevalence of HBV in this study was 7.8% (Table 1). According to the World Health Organization standards of high (≥ 8%), intermediate (2–7%), and low (< 2%) endemicity, the population falls within an intermediately high endemicity. This prevalence aligns with a study conducted in Ethiopia, which also reported a prevalence of 7.8% (Metaferia *et al.*, 2016). Similar studies in Nasarawa State have reported prevalences of 10.6% (Egbe, Ike, and Egbe, 2023), 9.4% (Innocent *et al.*, 2022), 8.0% (Ogbe *et al.*, 2020), 17.0% and 5.5% (Oti *et al.*, 2021), 9.7% (Pennap *et al.*, 2019), and 13.0% (Ya’aba, Owoseni, and Abioseabo, 2023). Variations in prevalence rates may result from differences in geographical locations, sampled populations, detection methods, study designs, and socio-cultural practices.

### Sociodemographic factors among the population

The sociodemographic results are presented in Table 2, revealing a higher prevalence in the age group 40–49 years. This finding contrasts with previous studies that reported varying prevalence across different age groups, such as 30–39 years (Ogbe *et al.*, 2020), 25–29 years (Pennap *et al.*, 2019), 11–19 years (Oti *et al.*, 2021), > 35 years (Deng *et al.*, 2023), ≥ 40 years (Kwadzokpui *et al.*, 2020), 60–69 years (Olayinka *et al.*, 2016), and 20–30 years (Okonkwo *et al.*, 2019). The high prevalence in the 40–49 age group may be influenced by cumulative lifetime exposure to risk factors or reduced access to early interventions during their youth. Lifestyle factors, particularly during late adolescence and early adulthood, can contribute to HBV occurrence across different age groups (Anka *et al.*, 2016). Additionally, traditional health practices, such as tonsil removal, have been linked to community-acquired HBV infection (Umer *et al.*, 2023).

A higher prevalence in males was also observed, aligning with previous studies (Edrees, Banafa, and Al-Awar, 2022; Okonkwo *et al.*, 2019; Tawiah *et al.*, 2022; Zafrin *et al.*, 2019). This could be due to higher engagement in high-risk behaviors, such as unprotected sexual activity, tattooing, traditional circumcision practices, and the use of unsterilized equipment by barbers (Degenhardt *et al.*, 2017). Additionally, biological differences may contribute, as females are more likely to produce anti-HBs antibodies against HBsAg than males (Matthews, Brown, and Goulder, 2022). However, some studies reported a higher prevalence in females (Amiwero *et al.*, 2017; Al-Matary and Al-Gashaa, 2022; Balegha, Yidana, and Abiiro, 2021; Pennap, Nuhu, and Oti, 2016; Upadhyay *et al.*, 2020), suggesting that varying cultural practices or regional factors may play a role.

Regarding education, participants with a secondary-level education showed the highest prevalence, consistent with other studies (Argaw *et al.*, 2022; Egbe, Ike, and Egbe, 2023; Kwadzokpui *et al.*, 2020). This may be due to limited awareness of transmission prevention or inadequate healthcare access in this group. However, other studies reported the highest prevalence among individuals with primary education (Ogbe *et al.*, 2020; Kwadzokpui *et al.*, 2020; Zafrin *et al.*, 2019), non-formal education (Metaferia *et al.*, 2016; Asaga, Chipago, and Ehi, 2019), or illiteracy (Ojara *et al.*, 2021), highlighting that education alone may not be sufficient to protect against infection without targeted public health interventions.

Unmarried or single individuals had the highest prevalence, potentially due to engagement in riskier lifestyles, such as multiple sexual partners or substance use, which are often associated with increased HBV transmission. This trend is supported by other studies (Alhassan *et al.*, 2021; Balegha, Yidana, and Abiiro, 2021; Isah *et al.*, 2020; Olayinka *et al.*, 2016; Ojara *et al.*, 2021; Oti *et al.*, 2021; Tawiah *et al.*, 2022). In contrast, some reports found higher prevalence among married individuals (Adoga *et al.*, 2019; Al-Matary and Al-Gashaa, 2022; Deng *et al.*, 2023; Okonkwo *et al.*, 2019; Tesfu, Habtemariam, and Belay, 2023; Ya’aba, Owoseni, and Abioseabo, 2023), suggesting that marital status may interact with other factors, such as social norms and healthcare access, affecting infection rates differently across populations.

Among occupations, public servants exhibited high prevalence, possibly due to misconceptions about transmission routes, with a focus on hospital-acquired infections while neglecting other risks like unsterilized medical tools in informal settings. Previous studies identified the highest prevalence among businessmen (Omatola, Onoja, and Agama, 2020), the unemployed (Fessehaye *et al.*, 2018), students, farmers (Oti *et al.*, 2021), and healthcare workers (Mboya *et al.*, 2023). This highlights the importance of tailoring awareness campaigns to different occupational settings to address specific risk factors.

Christianity was associated with the highest prevalence in this study, aligning with some reports (Mboya *et al.*, 2023; Kwadzokpui *et al.*, 2020), but contrasting with others that found a higher prevalence among Muslims (Alhassan *et al.*, 2021). This discrepancy may be explained by regional religious practices, cultural attitudes toward healthcare, or differences in healthcare access.

Finally, individuals earning ≥ N151,000 and above (100.0%) showed the highest prevalence with a significant association (p < 0.01). However, it must be noted that there was only 1 participant with a monthly income of ≥ N151,000. Kbrom *et al*. (2020) from Ethiopia reported 15.9% among individuals earning <1000 monthly, and Zafrin *et al*. (2019) from Bangladesh reported 46.4% among those earning <5000 monthly. The differences observed may be due to variations in sample populations, socio-economic status, and behavioural lifestyles of the respondents. income group could be attributed to their increased income level, potentially leading to engagement in high-risk behaviors that facilitate transmission, such as tourism, unhealthy sexual practices, and travel to endemic regions (Xiang *et al.*, 2022). This contrasts with studies that found the highest prevalence among low-income groups (Martyn *et al.*, 2023), where limited access to healthcare, preventive measures, and vaccinations may increase vulnerability to HBV infection.

### Association between the prevalence of HBV and risk factors

The chi-square (χ²) test results in Table 3 demonstrate associations between HBV prevalence and several risk factors. For family history of liver disease, 11 respondents (2.39%) with a family history of liver disease were infected, while 25 respondents (5.42%) without a family history of liver disease were also infected, but there was no association (p = 0.06). This finding is consistent with Weldebrhan, Berhe, and Tesfay (2023), Tesfa *et al*. (2021), and Adoga *et al*. (2019). However, this contrasts with Chan *et al*. (2022).

For those living with an HBV-infected person, 7 respondents (1.52%) who were infected reported living with an infected person, while 29 respondents (6.29%) who were infected had not lived with an HBV-infected person. There was no significant association (p = 0.87). Yildirim *et al*. (2014) reported that 11.5% of their study participants lived with infected individuals. This is possibly because in African cultures, close contact within family members is common, increasing the likelihood of viral transmission.

Regarding awareness of HBV status among respondents, 13 respondents (2.82%) who were infected with HBV were aware of their status, while 23 (4.99%) were unaware. There was a significant association between knowing one’s HBV status and HBV infection (p < 0.03). Otaigbe *et al*. (2023) reported 54.8%, Siraj, Fareed, and Mahajan (2016) found 56.52%, and Thote, Soyam, and Dhakate (2023) observed 35.7% awareness. This suggests that the association between knowing one’s HBV status and infection is possibly because people who know their status are more proactive about testing because they mostly have risk factors or symptoms that increase the likelihood of infection. Factors such as ignorance, privacy concerns, lack of awareness, personal choice, cultural influences, and fear of stigma and discrimination may explain why some respondents remain unaware of their status (Subic and Zoulim, 2018; Martyn *et al.*, 2023). Strengthening awareness and screening efforts, especially among high-risk populations, is essential.

For blood transfusion history, 3 respondents (0.65%) who were infected reported a history of blood transfusion, while 33 respondents (7.16%) had no such history. There was no association (p = 0.42). This study aligns with findings from Asaye *et al*. (2021) and Oti *et al*. (2021). These findings highlight the need for stronger national and state policies on blood transfusion to prevent HBV transmission via this route.

For contact with infected blood or bodily fluids, 5 respondents (1.08%) who were infected reported such contact, while 31 respondents (6.72%) had no contact. There was no association (p = 0.64). Umer *et al*. (2023) reported 28.0% prevalence, while Kayondo, Byamugisha, and Ntuyo (2020) observed 0.6%. Correia *et al*. (2001) noted that some respondents may have engaged in risky activities, leading to exposure to bloodborne pathogens. Preventive measures are necessary to reduce the risk of transmission in such high-risk settings.

For travel to HBV-endemic regions, 3 respondents (0.65%) who were infected had recently travelled to an endemic region, while 33 respondents (7.16%) had not travelled. Although there was no association (p = 0.60), studies by Falla *et al*. (2018) and Krarup, Rex, and Andersen (2020) indicate that travel and migration from low to high-endemic HBV regions increase the risk of exposure. Sonder, Rijckevorsel, and Hoek (2008) reported that 33% to 76% of travelers to HBV-endemic countries are at risk of infection. A study by Johnson, Leder, and Torresi (2013) found an incidence of 25 to 420 cases per 100,000 travelers per month to endemic regions (Bittaye *et al.*, 2019).

## Conclusion

From this study, it was observed that sociodemographic and risk factors are vital determinants of HBV in Lafia Metropolis, Nasarawa State, Nigeria. The prevalence in Lafia is intermediately high at 7.8%. Among the sociodemographic factors, the highest prevalence was observed in individuals aged 40–49 years (12.2%); males had a prevalence of 8.4%; singles had a prevalence of 8.6%; public servants had a prevalence of 15.8%; and those with a monthly income of N151,000 and above had a prevalence of 100% with a significant association (p < 0.01). A significant association (p < 0.03) was observed between HBV infection and knowing one’s HBV status. These findings are critical because they lead to a better understanding of the factors influencing HBV occurrence and help identify high-risk individuals. Thus, it is recommended that public health officials and policymakers develop targeted strategies for specific sociodemographic to address the HBV burden effectively in the study population.
